# A phase I trial of BNC105P and ibrutinib in patients with relapsed/refractory chronic lymphocytic leukemia

**DOI:** 10.1002/jha2.543

**Published:** 2022-08-11

**Authors:** Darcy B. Pooler, Dylan B. Ness, Alexey V. Danilov, Bridget M. Labrie, Tor D. Tosteson, Alan Eastman, Lionel D. Lewis, Frederick Lansigan

**Affiliations:** ^1^ Sections of Clinical Pharmacology and Hematology Oncology Department of Medicine Geisel School of Medicine at Dartmouth and Dartmouth‐Hitchcock Medical Center Lebanon New Hampshire USA; ^2^ The Dartmouth Cancer Center at Dartmouth‐Hitchcock Medical Center Lebanon New Hampshire USA; ^3^ Department of Hematology and Hematopoietic Cell Transplantation City of Hope Comprehensive Cancer Center Duarte California USA; ^4^ Department of Biomedical Data Science Geisel School of Medicine at Dartmouth Hanover New Hampshire USA; ^5^ Department of Molecular and Systems Biology Geisel School of Medicine at Dartmouth Hanover New Hampshire USA

1

Chronic lymphocytic leukemia (CLL) was projected to cause 4320 deaths in 2021 in the US [1]. Chemo‐immunotherapy regimens are commonly associated with unfavorable adverse events (AEs) [2, 3]. Targeted therapies such as the Bruton's tyrosine kinase (BTK) inhibitor ibrutinib are better tolerated and have become standard of care for CLL. Ibrutinib causes egress of CLL cells from their stromal niche leading to peripheral lymphocytosis [4]. While initially successful in treatment of naïve, relapsed/refractory, and high risk CLL (*deletion 17p*), most high risk patients experienced disease progression, indicating a need to improve BTK‐targeted therapy regimens [5, 6, 7, 8].

Microtubule inhibitors kill CLL cells ex vivo with dependence on c‐Jun N‐terminal kinase (JNK) activation and induction of NOXA [9]. Vincristine activated JNK but not NOXA in circulating CLL cells in vivo [10]. BNC105, a colchicine‐site microtubule inhibitor, was more potent at activating the JNK‐NOXA apoptotic cascade [11]. BNC105P, the prodrug of BNC105, had a monotherapy maximum tolerated dose (MTD) of 16 mg/m^2^ in solid tumor patients with on‐target polymerized tubulin reduction activity starting at 12.6 mg/m^2^ [12].

The study rationale was to target CLL cell egress from the stromal niche with ibrutinib and eliminate CLL cells in circulation with BNC105P to potentially obtain deep, long‐lasting responses [13]. We therefore conducted an open‐label, Phase Ib, dose escalation study of BNC105P and ibrutinib in patients with relapsed/refractory CLL (NCT03454165). Patients were enrolled at the Dartmouth Cancer Center using a Dartmouth College and Dartmouth‐Hitchcock Health Institutional Review Board approved protocol and written informed consent. Study patients provided written informed consent prior to the performance of any study procedures and given a copy of the signed written informed consent.

BNC105P (Bionomics Limited, Thebarton, Australia) was administered via IV infusion on days 1 and 8 (cycle 1) and days 8 and 15 (cycles 2–6) of 21‐day cycles. Ibrutinib 420 mg was self‐administered orally on days 1–21 (cycles 2–6). In drug combination cycles, ibrutinib was taken ≥30 min prior to BNC105P infusion. A standard 3 + 3 Phase I dose escalation design was used with planned BNC105P dose cohorts of 8 mg/m^2^ (cohort 1), 12 mg/m^2^ (cohort 2), and 16 mg/m^2^ (cohort 3). The primary study objective was to determine the MTD of BNC105P in combination with ibrutinib in CLL patients.

All patient inclusion and exclusion criteria for study NCT03454165 are detailed at https://clinicaltrials.gov/ct2/show/NCT03454165. Prior ibrutinib therapy was allowed but not required. Enrolled patient demographics are listed in Table 1. Clinical responses were assessed via computerized tomography scans. Modified IWCLL guidelines were used to categorize objective responses [14]. Dose limiting toxicities (DLTs) were defined as: all nonhematological toxicities of ≥ grade 3 except: reversible grade 3 nausea, vomiting or diarrhea; reversible asymptomatic grade 3–4 laboratory abnormalities; hematological toxicities: grade ≥ 3 febrile neutropenia; infection of grade 4, or accompanied by fever or fails to resolve within 7 days; failure of adequate recovery of neutrophils/platelets or neutrophil count to > 1000/platelets 50,000/mm^3^ (whichever is lower) within 14 days of the scheduled start day for cycle 2 or cycle 3. Grading of nonhematological toxicities utilized NCI CTCAE v4.03, while hematological toxicities were graded according to 1996 modified NCI‐WG criteria for CLL/SLL, updated in 2008 [14]. The MTD was defined as the dose at which no more than 1/6 of the subjects (16.7%) experience a DLT.

**TABLE 1:** Patient demographics and outcomes

Patient demographic		All cohorts *n* = 6	Cohort 1 (8 mg/m^2^) *n* = 3	Cohort 2 (12 mg/m^2^) *n* = 3^a^
**Sex**	Male	5	2	3
	Female	1	1	0
**Age**	Median (range)	72 (52–75)	71 (52–75)	73 (60–75)
	<65 y	2	1	1
	≥65 y	4	2	2
**BMI**	Median (range)	31.0 (21.2–38.2)	26.5 (21.2–32.9)	32.1 (29.9–38.2)
**Race**	White	6 (100%)	3 (100%)	3 (100%)
**Ethnicity**	Other (non‐Hispanic/Latino)	6 (100%)	3 (100%)	3 (100%)
**ECOG score**	0	1 (16.7%)	1 (33.3%)	0 (0%)
	1	5 (83.3%)	2 (66.7%)	3 (100%)
**Years since diagnosis**	Median (range)	9.5 (8–12)	9 (8–12)	10 (9–10)
**Prior therapies**	Median (range)	2.5 (1–8)	2 (1–8)	3 (2–3)
	Prior ibrutinib	2	1	1
**Patient outcomes**				
**# Cycles completed**	Median (range)	4.9 (1.7–6)	4.5 (2–5.3)	6 (1.7–6)
**# Days on study**	Median (range)	128 (49–178)	104 (100–154)	152 (49–178)
**Adverse events**	Median (range)	6 (2–12)	6 (6–12)	8 (2–12)
**Serious adverse events**	Total	2	1**^b^**	1**^a^** **^,^** **^c^**
**Dose limiting toxicity**	Total	1	0	1
**Best response achieved** **^d^**	PR	1**^a^**	0	1
	SD	3	2	1
	NE	2	1	1

^a^ One patient initially received BNC105P 12 mg/m^2^ monotherapy in cycle 1 and ibrutinib 420 mg monotherapy in cycle 2 (days 1–7) but because of a 25%–50% drop in platelets from baseline (from Gr1 →Gr2) was dose reduced to 8 mg/m^2^ BNC105P and ibrutinib 280 mg daily (as proscribed in the protocol but not a defining DLT) and subsequently tolerated this dose for six cycles with a partial remission.

^b^ This SAE was a patient who developed cryptococcal pneumonia following heavy self‐exposure to bat droppings (guano) while cleaning an attic. The SAE occurred on study cycle 3 day 7, which was outside the defined DLT time window for the study.

^c^ This SAE was sudden death (of unknown cause; no autopsy) of the patient described in footnote ^a^ who completed six cycles of 8 mg/m^2^ BNC105P + Ibrutinib 280 mg daily and was off combination study treatment and being treated only with ibrutinib monotherapy when the SAE occurred.

^d^ BMI, body mass index; ECOG, Eastern Cooperative Group; NE, not evaluable; PR, partial remission; SD, stable disease.

Three patients were treated in the 8 mg/m^2^ BNC105P plus 420 mg ibrutinib cohort (Cohort 1) without developing any DLTs. Subsequently three patients were treated with 12 mg/m^2^ BNC105P plus 420 mg ibrutinib (Cohort 2). One of the cohort 2 patients who initially received 12 mg/m^2^ of BNC105P in cycle 1 and 420 mg ibrutinib C2D1‐7 was subsequently dose reduced (as per protocol because he developed Gr 1→Gr2 thrombocytopenia, but this was not a defined DLT) to 8 mg/m^2^ BNC105P and 280 mg ibrutinib, which was tolerated for a further six cycles.

Patient outcomes, serious AEs (SAEs), and AEs are summarized in Table 1. One DLT of protracted grade 2 thrombocytopenia in cohort 2 was attributed to ibrutinib. Two SAEs occurred: one sudden death (unknown cause) occurred 10 days after completing study combination treatment while receiving ibrutinib monotherapy; one patient in cohort 1 developed cryptococcal pneumonia on C3D7 following heavy exposure to bat droppings (guano) at a timepoint beyond the defined DLT time window.

Treatment‐related AEs included (*n* of six patients): fatigue (3), anemia (3), constipation (2), bruising (2), hypertension (2), dizziness (1), neutropenia (1), thrombocytopenia (2), elevated ALT (2), lymphocytosis (1), hyponatremia (1), headaches (1), elevated AST (1), ankle edema (1), arthralgia (1), dysgeusia (1), and grade 2 protracted thrombocytopenia resulting in a DLT (1). All AEs were grade 1 or 2 except neutropenia (grade 4, baseline grade 3) and lymphocytosis (grade 3).

Of the six patients treated, one had a partial remission, three initially had stable disease (one sustained), and two were not evaluable for response. Absolute lymphocyte counts (ALCs) were measured (Figure 1A) with a marked increase (21%–433%) a week after ibrutinib therapy (first administered on C2D1). ALCs on C2D8 decreased following the BNC105P dosing by 6%–34% in five patients. Other peripheral blood mononuclear cells (PBMC) biomarkers were assessed by collecting whole blood pre‐dose, 0.5, 3, and 6 h post‐BNC105P infusion on C1D1 and C2D8, and preibrutinib dose on C2D1 as described previously [10, 11]. JNK was phosphorylated/activated (*n* = 6/6) and NOXA induced (*n* = 5/6) after C1D1 BNC105P treatment (Figure 1B,C). Apoptosis marker, cleaved PARP, was negligible (Figure 1D). Tubulin was destabilized and found in the soluble fraction of PBMCs after BNC105P treatment (*n* = 5/6) (Figure 1E).

**FIGURE 1:**
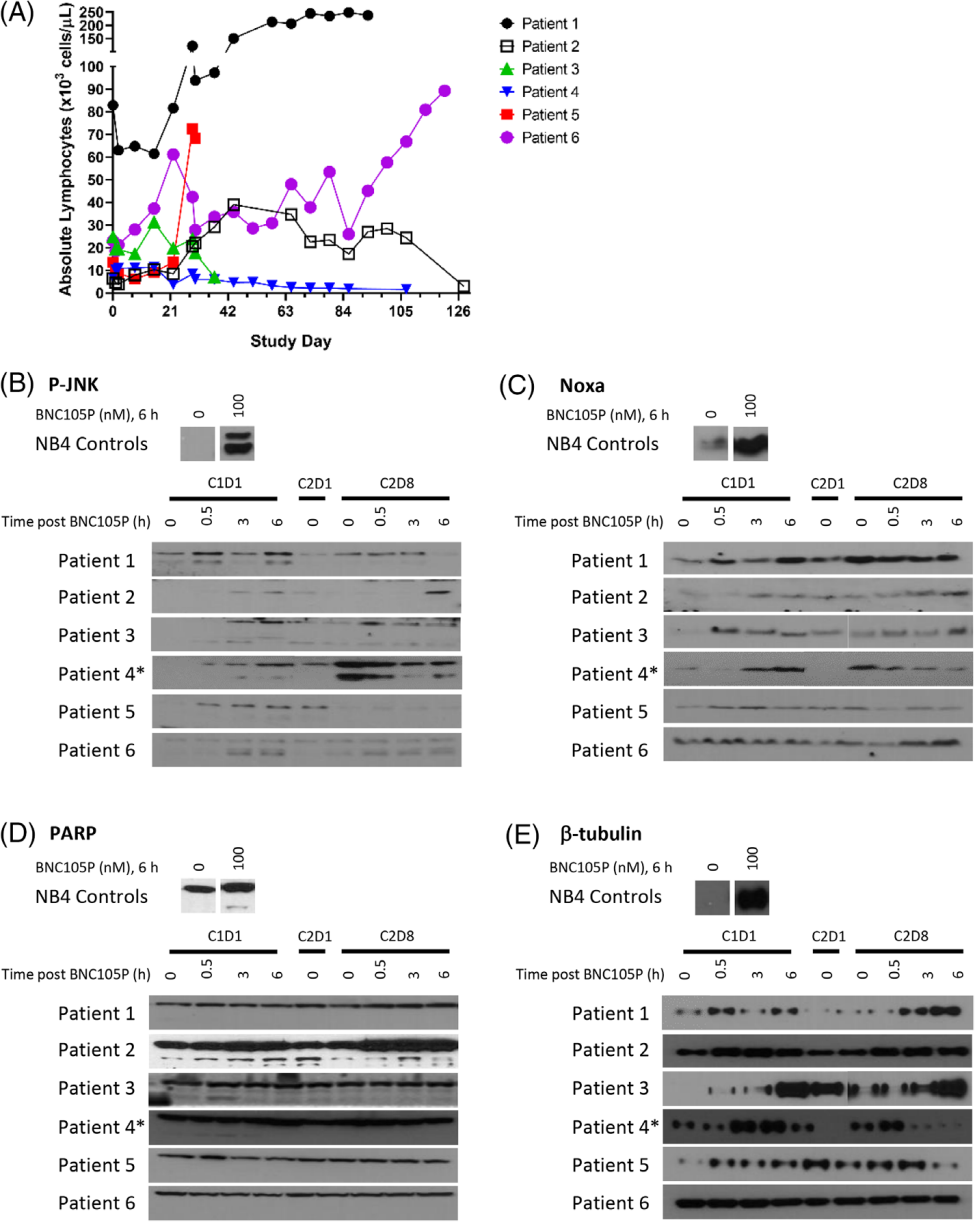
**In vivo biomarkers in PBMCs**: Absolute lymphocyte counts over time on study for each patient are shown in panel (A). Peripheral blood mononuclear cells (PBMCs) from each patient were collected pre‐dose (0 h), 0.5, 3, and 6 h post‐BNC105P infusion during C1D1 and C2D8, and pre‐ibrutinib on C2D1. Whole cell lysates from each sample were analyzed for P‐JNK (panel B), NOXA (panel C), PARP (panel D), and tubulin extracted lysates were analyzed for β‐tubulin (panel E). Bands for each protein were cropped from the same immunoblot to remove irrelevant data bands (e.g., empty lanes or replicate samples). NB4 cells were treated with 100 nM BNC105P for 6 h and served as a positive control cell line for all biomarkers for every patient blot with a representative NB4 control shown for each protein. *Patient 4 was dose reduced in cycle 2 and then tolerated six cycles of ibrutinib 280 mg/daily combined with BNC105P 8 mg/m^2^ on days 8 and 15 of each 21‐day cycle, with a partial remission

This study tested the novel combination of BNC105P with ibrutinib in patients with relapsed/refractory CLL. Unfortunately, the primary objective of this study (MTD determination) was not achieved due to study termination mandated by institutional policy, which defines that the rate of six patients enrolled over 3.5 years was excessively slow. This inadequate rate of enrollment was contributed to by the proven efficacy of the venetoclax plus rituximab combination [15] and Federal Drug Administration (FDA) approval of acalabrutinib.

The study drug combination at a dose of 8 mg/m^2^ IV BNC105P plus 420 mg PO ibrutinib was safe and well tolerated (no DLTs). Three patients were treated at the 12 mg/m^2^ IV BNC105P and 420 mg PO ibrutinib dose level. One patient was dose reduced as per protocol because of Gr 2 thrombocytopenia and was treated for 6 cycles with partial remission but having completed study treatment and while taking ibrutinib monotherapy suddenly died (unknown cause; no autopsy). A second patient had a DLT of protracted thrombocytopenia (Gr 2) and was unable to continue therapy beyond cycle 2. The third patient tolerated the Cohort 2 study doses for six cycles with initial stable disease.

The common major toxicities (>30%) were grades 1–2 anemia, fatigue, bruising, constipation, hypertension, and thrombocytopenia and are known adverse effects of ibrutinib therapy. Our study supports the utility of tubulin depolymerization as a biomarker for BNC105P on‐target activity. However, larger studies are required to establish the MTD, full safety profile, and apoptotic biomarker utility of this drug combination in CLL patients.

## AUTHOR CONTRIBUTIONS

*Concept and trial design*: DBP, AVD, TDT, AE, and LDL. *Clinical support*: BML, LDL, and FL. *Data collection and analysis*: DBP, DBN, BML, LDL, and FL. *Manuscript original draft*: DBP. *Data interpretation and final manuscript review*: Darcy B. Pooler, Dylan B. Ness, Alexey V. Danilov, Bridget M. Labrie, Tor D. Tosteson, Alan Eastman, Lionel D. Lewis, and Frederick Lansigan.

## CONFLICT OF INTEREST

DBP, DBN, BML, TDT, AE, FL have no competing interests to report in relation to this study. AVD is a paid consultant to Abbvie, AstraZeneca, BeiGene, Gamida, Genentech, Janssen, Morphosys, Pharmacyclics, and TG Therapeutics and has ongoing research funding from AstraZeneca, Bayer Oncology, Bristol Meyers Squibb, MEI Pharma, SecuraBio, TG Therapeutics, and Takeda Oncology. LDL is a paid consultant to G1 Therapeutics and 7 Hills Pharma LLC and receives support for clinical trials from Bristol Myers Squibb, AbbVie, and AstraZeneca.

2

## FUNDING INFORMATION

The Hopeman Foundation; Bench to Bedside Funding from The Dartmouth Cancer Center.

## Data Availability

Data may be requested from the corresponding author. All data provided are anonymized to respect the privacy of patients who have participated in the trial in line with applicable laws and regulations.
